# Supplementary material for: Left Atrial Functional Remodeling in Patients with Chronic Heart Failure Treated with Sacubitril/Valsartan
Source: J Clin Med. 2023 Jan 30;12(3):1086. doi: 10.3390/jcm12031086 (PMC9917469; doi:10.3390/jcm12031086)
Supplement: Supplementary file 1 [file jcm-12-01086-s001.zip › jcm-2117943-supplementary.pdf]

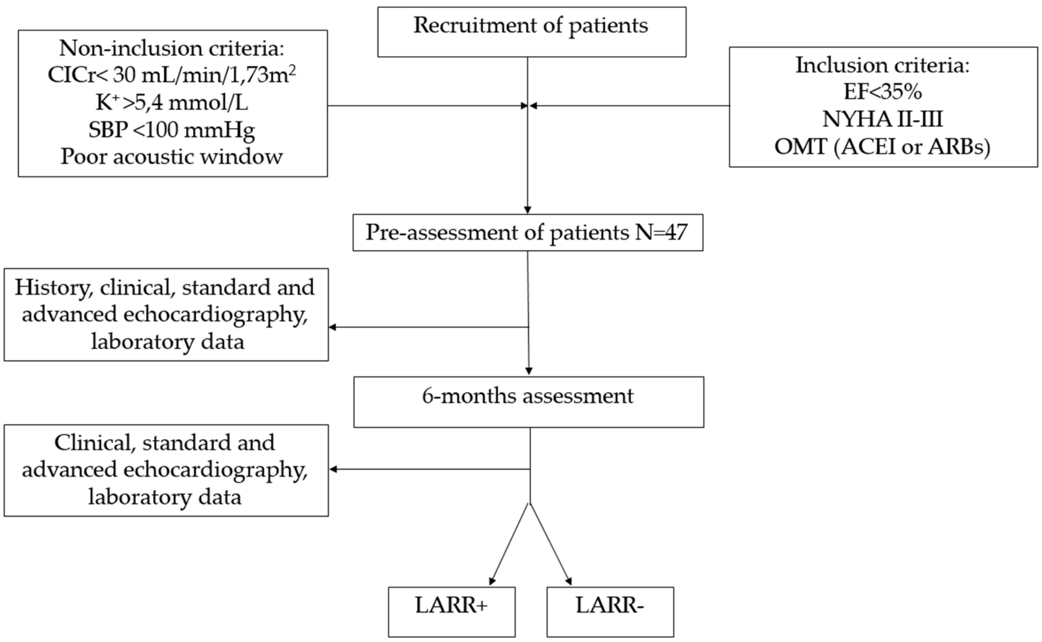

Figure S1. Study flow chart.

LVEF: left ventricular ejection fraction; NYHA: New York Heart Association; SBP: systolic blood pressure; eGFR: estimated glomerular filtration rate; Ace-i: angiotensin converting enzyme inhibitors; ARBs: angiotensin receptor blockers; CrCl: Creatinine Clearance; K^+: potassium.

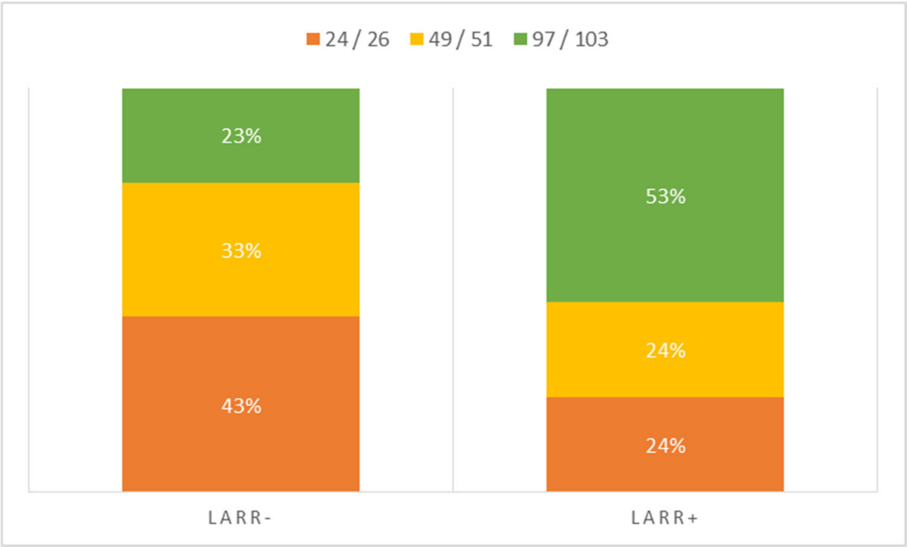

Figure S2. Sacubitril Valsartan dosage at 6-month follow up.

Table S1. Multivariable regression stepwise analysis with changes in LAVi.

Changes in LAVi	Beta	Std. Err.	b	Std. Err.	P-value
**Changes in LVEDV**	-0.4292	0.1315	-0.4244	0.1300	**0.0024**
**Changes in PALS**	-0.3283	0.1290	-0.1259	0.0495	**0.0153**
**Baseline LVEDD**	-0.2361	0.1315	-0.0061	0.0034	0.0808

LVEDV: left ventricular end-diastolic volume; PALS: peak atrial longitudinal strain; LVEDD: left ventricular end-diastolic diameter In bold p value < 0.05.
